# The Review of Hybridization of Transition Metal-Based Chalcogenides for Lithium-Ion Battery Anodes

**DOI:** 10.3390/ma16124448

**Published:** 2023-06-18

**Authors:** Lin-Hui Wang, Long-Long Ren, Yu-Feng Qin

**Affiliations:** 1 College of Information Science and Engineering, Shandong Agricultural University, Taian 271018, China; 2 College of Mechanical and Electronic Engineering, Shandong Agricultural University, Taian 271018, China

**Keywords:** transition metal, chalcogenides, anodes, lithium-ion batteries, hybridization, synergetic effect

## Abstract

Transition metal chalcogenides as potential anodes for lithium-ion batteries have been widely investigated. For practical application, the drawbacks of low conductivity and volume expansion should be further overcome. Besides the two conventional methods of nanostructure design and the doping of carbon-based materials, the component hybridization of transition metal-based chalcogenides can effectively enhance the electrochemical performance owing to the synergetic effect. Hybridization could promote the advantages of each chalcogenide and suppress the disadvantages of each chalcogenide to some extent. In this review, we focus on the four different types of component hybridization and the excellent electrochemical performance that originated from hybridization. The exciting problems of hybridization and the possibility of studying structural hybridization were also discussed. The binary and ternary transition metal-based chalcogenides are more promising to be used as future anodes of lithium-ion batteries for their excellent electrochemical performance originating from the synergetic effect.

## 1. Introduction

With the increasingly serious energy crisis, more and more attention has been paid to green energies, such as wind energy and solar energy [1,2]. To resolve the disadvantages of the intermittency and randomness of these green energies, the development of energy conversion and storage systems is becoming increasingly significant. Lithium-ion batteries (LIBs) have been widely explored and applied in portable electronics, electric vehicles, and high-power energy storage systems, on account of their long cycle life, and high specific capacity [3,4]. However, traditional carbon-based anodes are facing the challenges of improving the energy density and rate performance raised by high-power energy storage systems and artificial intelligence vehicles [5,6]. Despite the advantages of abundance, relatively low lithiation voltage, and high lithium storage for the alloying anodes, such as Si-based, Ge-based, and Sn-based materials, the large destructive effects of volumetric expansion during cycling and low intrinsic electronic conductivity prohibit the further application of these alloying anodes [7,8,9].

As a representative of the conversion reaction, transition metal chalcogenides, as one of the most promising alternatives to carbon-based anodes, have been extensively explored, exhibiting high specific capacity and outstanding rate performance [10,11,12]. For practical application, the drawbacks of volume expansion and low conductivity for these anode materials during the cycling process should be further resolved. Therefore, diverse nanostructures have been designed and prepared using many preparation methods. The nanostructures can increase the specific surface and decrease the intercalation path, and therefore increase the specific capacity of the transition metal chalcogenide anodes [13,14,15,16,17]. Cubic hollow CuS nano-boxes anodes exhibited superior cycle stability and high specific capacity at 20 C [13]. Synthesized by a sol-gel method, CuS nanorods achieved excellent cycle stability and high Coulombic efficiency [14]. Hu et al. synthesized CuS porous spheres and hollow spheres by a bubble template method, which indicated a promising alternative to carbon-based anodes for LIBs [15]. CuO anodes showed excellent electrochemical performance due to the unique morphology of the leaf-like nanoplates [16]. The core/shell-structured NiS anodes were prepared by the arc discharge method and exhibited good cyclability, high capacity, and outstanding rate capability [17]. Besides, carbon-based materials, such as graphene, reduced graphene oxide (rGO), and graphite, have been generally introduced to transition metal chalcogenides anodes. The introduction of carbon-based materials can enhance the cycle stability and conductivity of transition metal chalcogenide anodes to some extent [18,19,20,21,22,23]. In the rGO matrix, Fe_3_S_4_ nanoparticles were obtained by a one-pot hydrothermal method, showing excellent specific capacity and high conductivity [18]. The tubular mesoporous carbon materials were introduced to prepare Cu_2_S/C nanocomposites, which showed relatively good reversible capacity and rate capability [19]. CuS/CNTs nanocomposites were synthesized by a solvothermal method and exhibited good electrochemical performance due to the synergetic effect between carbon nanotubes and CuS [20]. CuO/graphene nanomaterials possessed an enhanced accommodation of lithium ions intercalation due to the synergetic effect of graphene [21]. Graphene with different structures was also introduced to Mn_3_O_4_ nanomaterials, which enhanced the conducive network and cycle stability of the anode materials [22,23].

In addition to the two above-mentioned solutions, the hybridization of transition metal-based chalcogenide anodes was reported, which could effectively enhance the electrochemical performance because of the synergetic effect of diverse components. The multifunctional component hybridization of the anode materials should be an effective way to accomplish excellent electrochemical performance for transition metal chalcogenides. It is worth noting that “hybridization” does not mean a simple mixture composed of two or more materials but a hybrid material with chemical links between each other in which the electronic structure of the hybrid material itself will be different from the mixture. Many works and reviews were reported based on the investigation of nanostructure designing and carbon-based material doping [13,14,15,16,17,18,19,20,21,22,23]. As far as we know, there are many studies on hybridization, but a summary of hybridization has not been reported. Generally, the hybridization can be divided into the hybridization between the transition metal and the transition metal chalcogenide, and the hybridization between different transition metal chalcogenides. In this review, we concentrate on the four types of hybridization, by which the individual advantages of each component could be strengthened and excellent electrochemical performance could be obtained. We also discuss the exciting problems of hybridization and the possibility of studying structural hybridization.

## 2. Hybridization between the Transition Metal and Transition Metal Chalcogenide

Because of the high conductivity and the catalytic property for the decomposition of Li_2_O, transition metal doping has been investigated, and the enhanced electrochemical performance of transition metal chalcogenide anodes was obtained. Generally, transition metal doping includes the hybridization of X and X-chalcogenides, and the hybridization of X and Y-chalcogenides, in which X and Y denote different transition metals, while X-chalcogenide and Y-chalcogenide represent different transition metal chalcogenides.

### 2.1. Hybridization of X and X-Chalcogenides

The transition metal could not only catalyze the reversible decomposition of Li_2_O but also help to establish the conductive network in the hybrid electrodes. The hybridization of a transition metal and the same metal-based chalcogenide was always achieved by accurately controlling the oxidation or reduction process.

**Figure 1:**
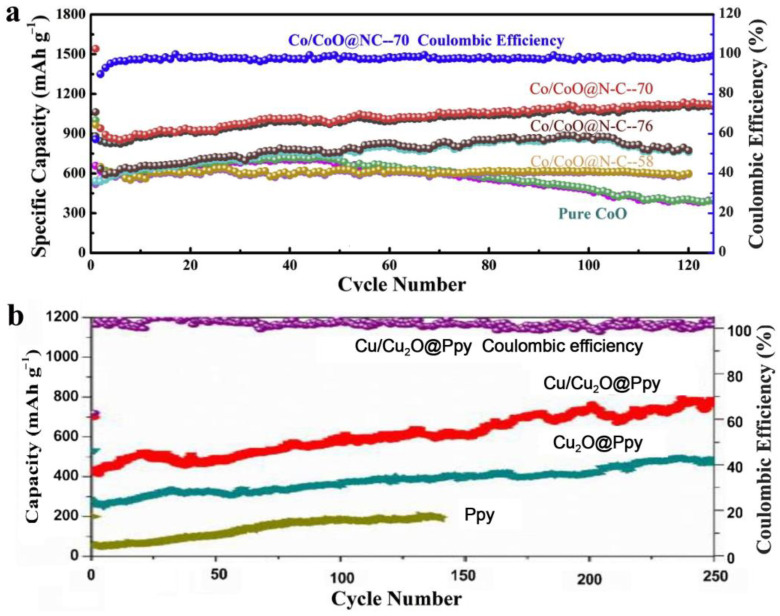
(**a**) Comparison of Co/CoO@N-C and pure CoO at 200 mA/g [26]. (**b**) Comparison of Cu/Cu_2_O@Ppy, Cu_2_O@Ppy, and pure Ppy at 100 mA/g [30].

Compared to pure CoO, the core-shell nanostructured Co@CoO exhibited superior electrochemical performance due to the existence of Co metal. According to their catalytic ability, the core Co metal nanoparticles could enhance the reversible decomposition of Li_2_O, while the outside Co metal portion could establish the conductivity network between the CoO anode materials [24]. Hao et al. prepared Co/CoO nanoparticles anchored on porous carbon cuboids by an impregnation method and consequent high-temperature treatment. Due to the synergetic effect between Co and CoO, as well as the unique hierarchical structure, the PCC-CoO*x* anodes showed high specific capability, long cycle life, and outstanding rate capability at high current density [25]. The Co/CoO@N-C nanocomposites were obtained by a facile chemical process and exhibited an enhanced initial capacity and a relatively high reversible specific capacity. The remarkable electrochemical performance originated from the increased conductivity and enhanced catalytic effect on Li_2_O, which were induced by heterogeneous composite doping [26]. Co_3_O_4_@CoO@Co nanocomposites were prepared by the hydrothermal method and following calcination treatment. The Co_3_O_4_@CoO@Co nanomaterials exhibited superior electrochemical and catalytic performance in the fields of LIBs and oxygen evolution reactions due to the modification of these components with each other [27]. Owing to the enhanced stability and improved conductivity achieved by the doping of metal Cu, the Cu_2_O/Cu nanowires exhibited greater remarkable cyclability and much higher capacity than conventional pure Cu_2_O [28]. The sub-microsphere structured Cu_2_O@Cu composites showed an outstanding rate performance and good cycling performance, which was ascribed to the synergistic effect between the volume-buffering capacity and the high electronic conductivity of metallic Cu in Cu_2_O@Cu composites [29]. The metallic Cu in Cu_2_O could act as a conducting way for electrons, a volume buffer during the cycling process, and a catalyst for the decomposition of Li_2_O. Wang et al. successfully synthesized core-shell structured Cu/Cu_2_O@Ppy nanowires by a one-step hydrothermal method, showing superior Li-storage performance caused by the nanowires’ structure and the introduction of Cu metal. In the anode materials, Cu metal could enhance the conductivity and the electrochemical reaction kinetics [30]. The nanostructured NiS/Ni electrode was prepared and showed high specific capacity and long cycle life resulting from the special porous structure and the formation of a conductivity network by Ni in the materials [31]. Zhu et al. explored a highly efficient and facile milling strategy to prepare Sn@SnO*x*/C nanocomposites. The Sn@SnO*x*/C anodes exhibited a stable reversible capacity after many cycles due to the amorphous microstructure and the conductive network established by the Sn nanoparticles [32]. The better electrochemical performance of a hybrid of X and X-chalcogenides than pure transition metal chalcogenide can be seen in Table 1 and Figure 1 [26,30].

### 2.2. Hybridization of X and Y-Chalcogenide

Compared to homoatomic metal doping, heteroatom hybridization could be easily accomplished by controlling the kinds and ratios of the chemical precursors. The hybrid of transition metal chalcogenide and heteroatoms tended to exhibit more remarkable electrochemical performance than pure transition metal chalcogenide.

Wang et al. found that the concentration of the doped Cu could regularly affect the morphologies and structures of the CoO/Cu nanocomposites. At a certain concentration, the CoO/Cu nanocomposites showed remarkable cycling stability to 1000 cycles and a high specific capacity of 580 mAh/g at 10 C, which are much better than pure CoO nanomaterials [33]. The outstanding electrochemical performance of the CoO/Cu anodes originated from the improved electron conductivity, enhanced catalytic function, and significant structure stability of CoO/Cu induced by Cu doping. The structure, morphologies, and electrochemical performance of a series of Ce-doped Mn_3_O_4_ nanocrystals with different Ce concentrations were thoroughly investigated. Mn_2.98_Ce_0.02_O_4_ anodes exhibited the highest capacity of 754.2 mAh/g up to 100 cycles, which was attributed to the bigger diffusion channels, more robust structure, and lower charge transfer impedance induced by the doping of Ce [34]. Krishnan et al. proposed a simple, environmentally friendly, and low-cost method to synthesize Mo-doped Cu_2_O:Mo with a porous microsphere structure. The remarkable electrochemical performance of the Cu_2_O:Mo anodes originated from the improvement of the specific surface area, electrical conductivity, Li-ion transports, and electrochemical kinetics induced by Mo doping [35]. The Zn-doped Co_3_O_4_ nanocomposites with a hollow concave structure displayed excellent rate capability and high specific capacities with stable cyclability. The outstanding electrochemical performance originated from the unique hollow concave structure and the defects induced by Zn doping, which could effectively improve the electronic structure of Co_3_O_4_ composites and then enhance the electrochemical reaction kinetics [36]. Wu et al. reported that the binary transition metal Cu_3.8_Ni was doped with CoO and MnO. The obtained Cu_3.8_Ni/CoO and Cu_3.8_Ni/MnO nanocomposites exhibited long cycle life, high energy density, and high power density, resulting from the establishment of the electron carrier path by Cu_3.8_Ni doping. [37].

Other metal-doped compound anodes for lithium-ion batteries were also reported. The physical properties and electrochemical performance of Mn-doped Co_2_(OH)_3_Cl xerogels depending on the Mn concentration were systematically investigated. Compared to pure Co_2_(OH)_3_Cl, the 4% Mn-doped Co_2_(OH)_3_Cl xerogels displayed a superior capacity and an outstanding rate capability, which could be mainly ascribed to the increased electric conductivity and charge transfer ability induced by Mn doping [38]. Through a ball milling activating technique, Li_4_Ti_5−x_Y_x_O_12_ anode materials were obtained by Y doping to Li_4_Ti_5_O_12_ and exhibited fast diffusion of lithium ions, outstanding rate capability, and excellent long-cycle stability. The introduction of hetero valence Y can establish a new charge balance with the spinel structure unchanged, by which the Li diffusion coefficient, electrical conductivity, and electrochemical performances were improved [39]. The better electrochemical performance of the hybrid of X and Y-chalcogenide than pure transition metal chalcogenide was summarized in Table 2.

## 3. Hybridization between Different Transition Metal Chalcogenides

Recently, many binary and ternary transition metal chalcogenides have been prepared and exhibited outstanding electrochemical performance. The hybridization of transition metal chalcogenides could promote the advantages of each chalcogenide and suppress the disadvantages of each chalcogenide. Therefore, the excellent electrochemical performance of a hybrid of transition metal chalcogenides could be obtained on account of the synergetic effect.

### 3.1. Hybridization of X-Chalcogenide and X-Chalcogenide

Due to the multivalent state of the transition metals, the two phrases of X-chalcogenide in the above title represent two different X-based chalcogenides, such as CoO and Co_3_O_4_, MnO and Mn_3_O_4_, CuO and Cu_2_O. Therefore, the hybridization of the same transition metal-based but different chalcogenides is discussed in this part.

Zhu et al. prepared dandelion-like CoO/Co_3_O_4_/C nanocomposites by a hydrothermal method and the following unique combustion method. The CoO/Co_3_O_4_/C anodes exhibited stable and high capacity at different current densities [40]. Prepared by a hydrothermal reaction and a subsequent calcination and reduction process, porous Co_3_O_4_@CoO nanosheet anodes showed an outstanding rate capability even at 5 A/g and an excellent specific capacity [41]. Li et al. synthesized Co(OH)_2_/Co_3_O_4_/Co nanoparticles derived from the EDTA-Co(II) sodium complex. The anode materials exhibited a high reversible capacity, good cycling stability, and high-rate performance, which originated from the porous structure, the presence of conductive Co, and especially the synergetic effect of Co(OH)_2_ and Co_3_O_4_ [42]. The MnO/Mn_3_O_4_@NC composites with a porous structure were prepared and used as anodes of LIBs. The relatively high electrochemical performance resulted from the special porous structure and the co-doping of N and Mn [43]. After 630 cycles, the MnO/Mn_3_O_4_/SeO*_x_* (*x* = 0, 2) hybrid anodes exhibited a reversible specific capacity of up to 1007 mAh/g at 3 A/g, resulting from the synergetic effect between MnO and Mn_3_O_4_ phases [44]. Grown on conductive collectors of β-NiS@Ni_3_S_2_, the bind-free anodes of NiO nanosheet arrays exhibited outstanding lithium storage properties owing to the special heterostructures and the synergetic effect of the Ni_3_S_2_, β-NiS, and NiO components [45]. The CuS/Cu_1.8_S nanocomposite anodes showed a better electrochemical performance than that of most of the previous reports, owing to the nano-size structure and the assistance of the Cu_1.8_S component [46].

**Figure 2:**
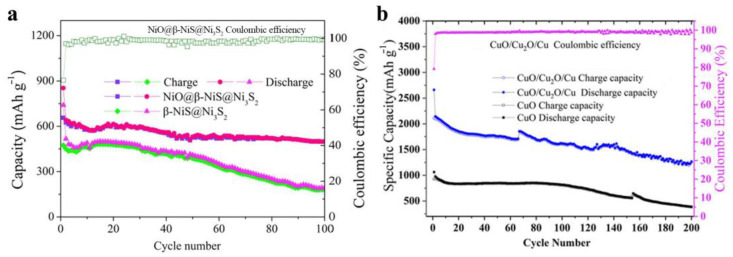
(**a**) Comparison of the NiO@β-NiS@Ni_3_S_2_ and β-NiS@Ni_3_S_2_ electrodes at 500 mA/g [45]. (**b**) Comparison of CuO/Cu_2_O/Cu and CuO nanowires nanocomposites at 200 mA/g [49].

Yolk–shell structured Cu_2_O@CuO nanocomposites were prepared by a multistep chemical method at room temperature. The anode nanocomposites exhibited superior cycling stability and rate capability with a reversible capacity of up to 854 mAh/g at 0.1 C after 200 cycles, which could be mostly attributed to the improved conductivity induced by the spur-CuO bridge and CuO shell [47]. Quaternary Cu_2_O/CuO/Cu/Carbon-polymer composite fibers were obtained and exhibited good rate performance, excellent cycle stability, and high capacity due to the fiber structure and the combination of metal and metal oxides [48]. Compared to pure CuO nanowires, the CuO/Cu_2_O/Cu exhibited much better electrochemical performance, which was ascribed to the synergetic effect of the ternary composites [49]. The outstanding electrochemical performance of the CuO/Cu_2_O/C anodes with uniform spherical morphology was attributed to the synergetic effect of the two-component CuO/Cu_2_O, porous structure, and conducting carbon coating [50]. Ternary CuO/Cu_2_O/Cu composites were designed and prepared by a method of electrodeposition and following calcination in air. The obtained bind-free CuO/Cu_2_O/Cu anodes displayed a high capacity, which originated from the cypress-like structure and the synergetic effect between the conductive Cu and the active Cu oxides [51]. Used in lithium/sodium-ion batteries, the CuO/Cu_2_O anodes anchored in graphite matrix exhibited high capacity and cycling stability, mainly due to the synergetic effect of the CuO and Cu_2_O nanoparticles [52]. Other CuO/Cu_2_O nanocomposite anodes with various structures were prepared by different methods and exhibited relatively outstanding electrochemical performance. The synergetic effect of the components of CuO and Cu_2_O plays an important role in enhancing the rate capability, specific capacity, and cycling stability of these nanocomposite anodes [53,54,55]. From Figure 2 and Table 3, we could draw the conclusion that the hybridization of the homoatomic transition metal chalcogenides can effectively enhance the electrochemical performance of a single pure transition metal chalcogenide [45,49].

### 3.2. Hybridization of X-Chalcogenide and Y-Chalcogenide

The hybridization of different transition metal-based chalcogenides, i.e., X-metal chalcogenide and Y-metal chalcogenide, was widely investigated and could be easily achieved by many different preparation methods. The electrochemical performance of the composites of binary or ternary transition metal-based chalcogenides was almost better than that of each transition metal-based chalcogenide because of the synergetic effect of the components. The hybridization of transition metal chalcogenides could effectively enhance the advantages of a single component at certain concentrations.

A hierarchical Fe_3_O_4_/CuO hybrid wire showed high capacities, excellent rate capabilities, and ultrafast diffusion of Li ions, which was much better than the pure Fe_3_O_4_ or CuO nanomaterial, respectively, originating from the intelligent integration of the Fe_3_O_4_ and CuO and the unique nanowires structure [56]. Due to the synergetic effect between Mn_3_O_4_ and Fe_3_O_4_ and the unique flower-like structure, the Mn_3_O_4_/Fe_3_O_4_ nanocomposites showed superior lithium ion storage ability than pure Fe_3_O_4_ or Mn_3_O_4,_ respectively [57].

Porous Co_3_O_4_/CuO composites also displayed better cycling stability and higher capacities than pure Co_3_O_4_ or CuO electrodes because of the synergetic lithium storage effect of both Co_3_O_4_ and CuO, as well as the porous hierarchical structure [58]. The ratios of Co and Cu could affect the morphologies, structures, and further electrochemical performance of the porous Co_3_O_4_/CuO composites, which indicated the vital role of hybridization to adjust electrochemical properties [58]. Directly grown on Cu foam, the heterostructured CoO/SiO_2_ exhibited high reversible capacity and good rate capability mainly because of the excellent synergetic effect between the two active materials of CoO and SiO_2_, and the enhanced conductivity of the 3D copper foam [59]. The binary ZnO/CoO mesoporous microspheres with carbon shells were prepared and delivered a good rate capability and high reversible capacity of 1457 mAh/g after 500 cycles, which was ascribed to the synergetic effect of the two components [60]. Composed of cubic Co_3_O_4_ and rhombus NiO, the flower-like NiO/Co_3_O_4_ composites exhibited a low charge transfer resistance and a high initial discharge capacity, indicating the improved conductivity and electrochemical reaction originated from the two metal cations [61]. For the hybrid nanofibrous Co_3_O_4_/TiO_2_, the initial discharge capacity was higher than the theoretical capacity of pure Co_3_O_4_ at 50.6% Co_3_O_4_ mass content [62]. The reversible capacity of amorphous hybridized SnO_2_/Co_3_O_4_ nanoflakes with a certain ratio of Sn and Co was much better than that of pure Co_3_O_4_ and SnO_2_ electrodes, respectively, which was attributed to the different potentials and working mechanisms of the SnO_2_ and Co_3_O_4_ [63]. Prepared by facile solvothermal and freeze-dring methods, the 3D structured CoO/ZnO nanoclusters anodes delivered a much higher specific capacity, which indicated an improved electrochemical performance by the synergetic effect of CoO and ZnO components [64]. The Co_3_O_4_/CeO_2_ heterostructure nanocomposites with different molar ratios of Co/Ce were prepared by a facile microwave assistance method. The excellent cycling stability and high reversible capacity of the 5Co_3_O_4_/CeO_2_ anodes resulted from the appropriate hybridization of Co_3_O_4_ and CeO_2_ [65]. Synthesized by a series of chemical reaction processes, the hierarchical mesoporous structured CoO@TiO2@C anodes exhibited much better electrochemical performance than that of pure CoO anodes, which indicated the enhanced effect of the robust TiO_2_ and amorphous carbon shells [66]. For the NiO/CoO nanoneedles, the enhancement of the electrochemical performance mainly resulted from the structural stability and the volume accommodation because of the interspace between NiO-CoO nanoneedles [67].

**Figure 3:**
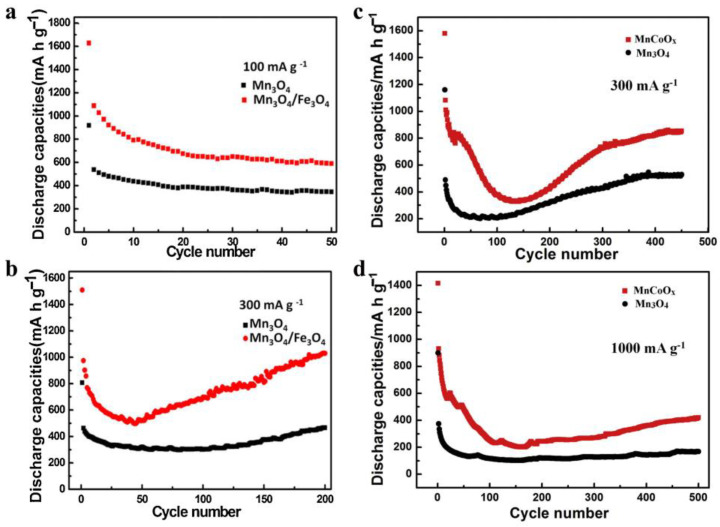
Comparison of the Mn_3_O_4_/Fe_3_O_4_ and Mn_3_O_4_ electrodes at 100 mA/g (**a**) and 300 mA/g (**b**) [57]. Comparison of the MnCoO_x_ anode and pure Mn_3_O_4_ material at 300 mA/g (**c**) and 1000 mA/g (**d**) [63].

**Figure 4:**
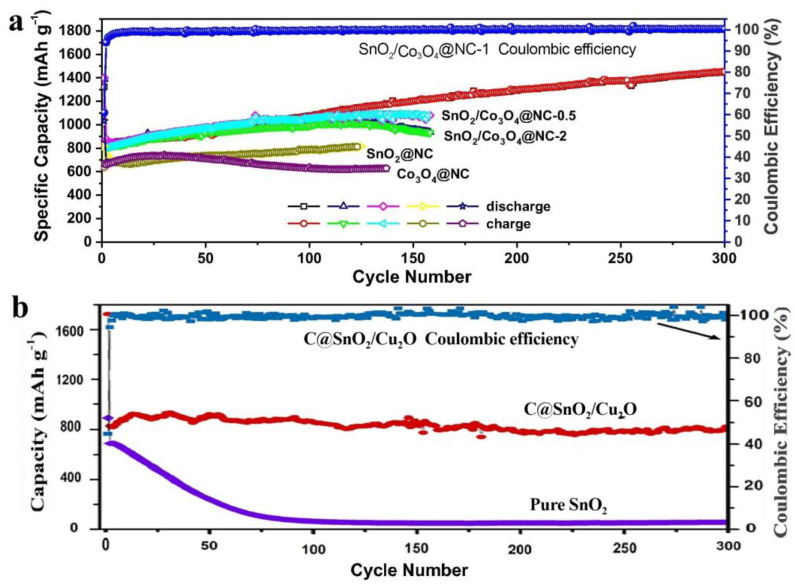
(**a**) Comparison of the SnO_2_/Co_3_O_4_@NC, SnO_2_@NC, and Co_3_O_4_@NC electrodes at 200 mA/g [72]. (**b**) Comparison of cycling performance of the C@SnO_2_/Cu_2_O electrode and pure SnO_2_ electrode at 0.5 C [73].

The core-shell CuO@ZnO composites with different contents of ZnO were synthesized by a chemical process of depositing ZnO on the CuO surface. Compared to pure CuO materials, the specific CuO@ZnO-6.5% anodes showed much superior electrochemical performance [68]. Cu_7.2_S_4_/C was introduced to form the core-shell Cu_7.2_S_4_/C@MoS_2_ nanocomposites, which exhibited long-cycle stability and high specific discharge capacity owing to the special oxidation states of Cu and the improved conductivity by the Cu-rich Cu_7.2_S_4_ component [69]. ZnO was introduced to suppress the agglomeration and migration of Cu_2_O or Cu particles during the carbonization process, and the molar ratios of Zn to Cu can directly affect the size of the bimetallic oxides. At a certain concentration, the carbon-confined Cu_2_O/ZnO showed a reversible capacity of up to 476 mAh/g [70]. Porous Cu_2_O/Mn_3_O_4_ hetero-nanosheets exhibited good cycling stability, high reversible capacity, and competitive rate capability because of the synergetic effect of the two components [71]. The flower-like C@SnO_2_/Cu_2_O nanosheet cluster anodes showed much better storage capacity, rate performance, and cycle stability than pure SnO_2_ anodes because of the carbon layer, the novel structure, and the synergetic effect between the nanosized SnO_2_ and Cu_2_O [72]. Prepared by a facile dealloying process of MnCoAl, MnCoO_x_ microspheres anodes exhibited better rate capability, superior cycling stability, and higher capacities than those of pure Mn_3_O_4_ anodes [73]. Figure 3 exhibited the better electrochemical performance of the hybrid of Mn_3_O_4_ than that of pure Mn_3_O_4_ [57,63], while Figure 4 showed the better electrochemical performance of the hybrid of SnO_2_ than that of each pure component [72,73], indicating the enhanced electrochemical performance by the hybridization of different transition metal-based chalcogenides.

Due to the unique architecture and the synergetic effect between the two composites, the bind-free Co(OH)_2_@MnO_2_ nanosheets grown on Ni foam showed outstanding electrochemical performance [74]. Multilayer amorphous SnO_2_ and TiO_2_ thin films displayed good electrochemical performance due to the heterogeneous structure and the two components. The SnO_2_/TiO_2_ nanocomposite integrated the advantages of the long cycle life of TiO_2_ and the high specific capacity of SnO_2_, indicating the enhanced effect of hybridization [75]. The hybrid of transition metal selenides was also synthesized by different methods and exhibited superior electrochemical performance. The CoFeSe/NC anodes exhibited better electrochemical performances (775 mAh g^−1^ after 50 cycles at 0.2 A g^−1^ and 423 mAh g^−1^ at 3 A g^−1^ up to 1000 cycles) and dynamic kinetics than the single-metal selenides, profiting from the synergistic effect of the multi-metal components, and well-retained integrated architecture [76]. Zhang and Pang demonstrated the excellent electrochemical performance of the hybrid of transition metal selenides in energy storage systems, such as lithium-ion batteries, sodium-ion batteries, and supercapacitors, respectively [77,78]. The better electrochemical performance of the hybrid of X-chalcogenide and Y-chalcogenide than pure transition metal chalcogenide was summarized in Table 4.

## 4. Discussion and Prospect

As is shown in Table 1, Table 2, Table 3 and Table 4 and Figure 1, Figure 2, Figure 3 and Figure 4, the electrochemical performance of hybrid anode materials was always better than that of pure anode materials. Hybridization is an effective method to improve the electrochemical performance of transition metal chalcogenides. Some researchers ascribe the enhanced mechanism of hybridization to the synergetic effect of the different components [64,65,66], while some researchers believe the interspace of the different phases plays an important role [67]. However, the details of the enhanced mechanism or the synergetic effect have not been further investigated. Based on the experimental results, a suitable theoretical calculation model should be established, and the enhanced mechanism should be deeply studied by using classical molecular dynamics and first-principle calculations in detail. If the interaction of different atoms or different phases is explored clearly, transition metal chalcogenides with excellent electrochemical performance will be prepared and used as anodes practically.

Till now, the study of hybridization has always focused on the hybridization of different crystal components. The study of amorphous/amorphous and amorphous/crystal hybridization has not been widely explored. The amorphous materials have unique features, such as an isotropic nature and more defects, which could provide more active sites and accommodate the volume expansion during the charge-discharge process [79]. Many molecular dynamics simulations and experimental results indicate that the electrochemical performance of amorphous anodes is better than that of crystal anodes [80,81]. The electrochemical performance of amorphous and crystalline Sn@C composites was compared, and the better electrochemical performance of amorphous anodes was ascribed to the unique features of amorphous structures [82]. A novel amorphous MoS_2_/MoO_3_ anode exhibited high specific capacity and cycling stability [83]. Compared to the crystal CoS anode, amorphous CoS exhibited much higher specific capacity and rate capability [10]. The amorphous CoO buffer was introduced to accommodate the volume expansion of CoO nanosheets, by which the CoO nanosheets/CoO film exhibited better electrochemical performance than CoO nanosheets [12]. Based on the unique features of amorphous structures, it is necessary to study structural hybridization combined with component hybridization, such as amorphous/amorphous and amorphous/crystal, which could accommodate the volume expansion and then improve the electrochemical performance of transition metal chalcogenides.

## 5. Conclusions

Due to the synergetic effect, hybridization is an effective way to enhance the electrochemical performance of transition metal chalcogenides as anodes in lithium-ion batteries. The hybridization could be divided into the hybridization between the transition metal and transition metal-based chalcogenide, and the hybridization between different transition metal-based chalcogenides. The electrochemical performance of a hybrid of transition metal chalcogenides is always better than that of pure transition metal chalcogenides. Though excellent electrochemical performance was obtained through the hybridization by many different preparation methods, the enhanced mechanism should be deeply investigated using classical molecular dynamics and first-principle calculations in the future. According to the unique features of amorphous structures, structural hybridization combined with component hybridization, such as amorphous/amorphous and amorphous/crystal, should be further investigated to improve the electrochemical performance. After a thorough understanding of the enhanced mechanism of the hybridization, we believe that a well-designed hybrid of transition metal-based chalcogenides is more promising to be used as future anodes of lithium-ion batteries.

## Figures and Tables

**Table 1:** The electrochemical performance comparison of X and X-Chalcogenides hybrid materials and pure materials.

Hybrid Materials Capacity [mAh g^−1^]		Pure Materials Capacity [mAh g^−1^]		Current [mA g^−1^]	Refs.
Co@CoO	800 (50 cycles)	CoO	200 (50 cycles)	50	[24]
PCC-CoO_x_	618 (500 cycles)	CoO	452 (500 cycles)	500	[25]
Co/CoO@N-C	1115 (125 cycles)	CoO	718 (125 cycles)	200	[26]
Cu/Cu_2_O@Ppy	787 (250 cycles)	Cu_2_O@Ppy	200 (140 cycles)	100	[30]
Sn@SnO_x_/C	430 (200 cycles)	Sn-SnO_2_-C	300 (200 cycles)	250	[32]

**Table 2:** The electrochemical performance comparison of X and Y-Chalcogenide hybrid materials and pure materials.

Hybrid Materials Capacity [mAh g^−1^]		Pure Materials Capacity [mAh g^−1^]		Current [mA g^−1^]	Refs.
Co_0.95_Cu_0.05_O	800 (80 cycles)	CoO	500 (200 cycles)	0.5 C	[33]
Mn_2.98_Ce_0.02_O_4_	754.2 (100 cycles)	Mn_3_O_4_	306 (100 cycles)	100	[34]
Cu_2_O:Mo	1122 (100 cycles)	Cu_2_O	820 (100 cycles)	100	[35]
Mn/Co_2_(OH)_3_Cl	1377 (50 cycles)	Co_2_(OH)_3_Cl	277 (50 cycles)	100	[38]

**Table 3:** The electrochemical performance comparison of X-Chalcogenide and X-Chalcogenide hybrid materials and pure materials.

Hybrid Materials Capacity [mAh g^−1^]		Pure Materials Capacity [mAh g^−1^]		Current [mA g^−1^]	Refs.
Co_3_O_4_@CoO	892 (200 cycles)	Co_3_O_4_	425 (200 cycles)	500	[41]
Co(OH)_2_/Co_3_O_4_/Co	543 (300 cycles)	Co_3_O_4_/Co	200 (100 cycles)	100	[42]
MnO/Mn_3_O_4_/SeO*_x_*	1007 (630 cycles)	Mn_3_O_4_/SeO*_x_*	544 (300 cycles)	1000	[44]
NiO@NiS@Ni_3_S_2_	498 (100 cycles)	NiS@Ni_3_S_2_	175 (100 cycles)	500	[45]
Cu_2_O@CuO@RGO	516 (60 cycles)	Cu_2_O@RGO	282 (60 cycles)	0.1 C	[47]
CuO/Cu_2_O/Cu	1266 (200 cycles)	CuO	385 (200 cycles)	50	[49]
CuO/Cu_2_O/Cu	534 (100 cycles)	Cu_2_O/Cu	297 (100 cycles)	100	[51]

**Table 4:** The electrochemical performance comparison of X-Chalcogenide and Y-Chalcogenide hybrid materials and pure materials.

Hybrid Materials Capacity [mAh g^−1^]		Pure Materials Capacity [mAh g^−1^]		Current [mA g^−1^]	Refs.
Fe_3_O_4_/CuO	953 (100 cycles)	Fe_3_O_4_	260 (100 cycles)	820	[56]
Fe_3_O_4_/CuO	953 (100 cycles)	CuO	559 (100 cycles)	820	[56]
Mn_3_O_4_/Fe_3_O_4_	600 (50 cycles)	Mn_3_O_4_	400 (50 cycles)	100	[57]
Co_3_O_4_/CuO	800 (80 cycles)	Co_3_O_4_	246 (80 cycles)	100	[58]
Co_3_O_4_/CuO	800 (80 cycles)	CuO	588 (80 cycles)	100	[58]
CoO/SiO_2_	1251 (250 cycles)	CoO	752 (50 cycles)	500	[59]
ZnO-CoO@NC	1457 (500 cycles)	ZnCo_2_O_4_	889 (400 cycles)	200	[60]
SnO_2_/Co_3_O_4_	1450 (300 cycles)	Co_3_O_4_	625 (135 cycles)	200	[63]
SnO_2_/Co_3_O_4_	1450 (300 cycles)	SnO_2_	800 (125 cycles)	200	[63]
Co_3_O_4_/CeO_2_	1131 (80 cycles)	Co_3_O_4_	539 (100 cycles)	100	[65]
CoO@TiO_2_@C	1136 (200 cycles)	CoO	17 (100cycles)	500	[66]
CoO@TiO_2_@C	1136 (200 cycles)	TiO_2_	94 (100 cycles)	500	[66]
CuO@ZnO	400 (500 cycles)	CuO	200 (130 cycles)	0.2 C	[68]
Cu_7.2_S_4_/C@MoS_2_	1415 (42 cycles)	MoS_2_	254 (42 cycles)	200	[69]
Cu_7.2_S_4_/C@MoS_2_	1527 (340 cycles)	Cu_7.2_S_4_/C	294 (68 cycles)	1000	[69]
Cu_2_O/Mn_3_O_4_	792 (350 cycles)	CuMnO_x_	238 (120 cycles)	2500	[71]
C@SnO_2_/Cu_2_O	814 (300 cycles)	SnO_2_	64 (100 cycles)	0.5 C	[72]
MnCoO_x_	860 (450 cycles)	Mn_3_O_4_	550 (450 cycles)	300	[73]
MnCoO_x_	420 (500 cycles)	Mn_3_O_4_	168 (500 cycles)	1000	[73]
Co(OH)_2_@MnO_2_	700 (150 cycles)	Co(OH)_2_	575 (150 cycles)	250	[74]
SnO_2_/TiO_2_	175 μAhcm^−2^ (200 cycles)	SnO_2_	10 μAhcm^−2^ (200 cycles)	13.8 μAcm^−2^	[75]

## Data Availability

Not applicable.
